# The Pneumococcal Divisome: Dynamic Control of *Streptococcus pneumoniae* Cell Division

**DOI:** 10.3389/fmicb.2021.737396

**Published:** 2021-10-18

**Authors:** Nicholas S. Briggs, Kevin E. Bruce, Souvik Naskar, Malcolm E. Winkler, David I. Roper

**Affiliations:** ^1^School of Life Sciences, University of Warwick, Coventry, United Kingdom; ^2^Department of Biology, Indiana University Bloomington, Bloomington, IN, United States; ^3^Department of Infectious Disease, Imperial College London, London, United Kingdom

**Keywords:** *Streptococcus pneumoniae* (pneumococcus), cell division, peptidoglycan (PG) synthesis, FtsZ, antibiotic resistance

## Abstract

Cell division in *Streptococcus pneumoniae* (pneumococcus) is performed and regulated by a protein complex consisting of at least 14 different protein elements; known as the divisome. Recent findings have advanced our understanding of the molecular events surrounding this process and have provided new understanding of the mechanisms that occur during the division of pneumococcus. This review will provide an overview of the key protein complexes and how they are involved in cell division. We will discuss the interaction of proteins in the divisome complex that underpin the control mechanisms for cell division and cell wall synthesis and remodelling that are required in *S. pneumoniae*, including the involvement of virulence factors and capsular polysaccharides.

## Introduction

Bacterial cell division is a fundamental and highly regulated process. It is heavily reliant on the coordination of peptidoglycan (PG) synthesis outside the cell membrane with molecular events occurring inside the cell, such as chromosome replication and separation, as well as membrane invagination and septation (Egan and Vollmer, 2013). Coordination between these components is critical for successful cell division, as many of the control checkpoints rely on signals from the cytoplasmic face of the membrane being used to regulate events outside the cell (Trusca et al., 1998; Haeusser and Margolin, 2016; Willis and Huang, 2017; Egan et al., 2020). This includes degradation or remodelling of the “old” cell wall PG sacculus outside the cell membrane and the creation of new PG for daughter cells (Typas et al., 2012; Massidda et al., 2013; Egan et al., 2020). PG is a three-dimensional mesh of glycan strands crosslinked together by short peptide stems [reviewed in Vollmer et al. (2019) for *Streptococcus pneumoniae*, and in Vollmer et al. (2008) more generally]. PG provides cell shape and resistance to turgor pressure and is a distinctive structural and chemical feature of bacteria, making it a widely used antibiotic target (Macheboeuf et al., 2006; den Blaauwen et al., 2014; Bush and Bradford, 2016). The biosynthesis of PG is a complex process performed by dedicated enzymes and protein complexes that begins in the cytoplasm and continues outside the cell membrane (Typas et al., 2012). Disruption of PG biosynthesis by inhibition of the enzymes responsible for its formation or sequestration of a key substrate intermediate, can be lethal to bacteria and has been the basis for life saving β-lactam chemotherapy for decades (Bush and Bradford, 2016).

Additionally, proper growth and division requires the coordinated remodelling of existing PG by dedicated PG hydrolases that degrade and modify its polymeric form, thus enabling growth and division. At present there is a significant lack of understanding of this process, particularly with regards to the required coordination of cell division with new cell wall PG biosynthesis. This is a subject of fundamental biological interest and may also provide further insight into future antimicrobial disruption of this vital process (Macheboeuf et al., 2006; den Blaauwen et al., 2014; Fisher and Mobashery, 2020). Previous microbiological investigation of bacterial cell morphology, encompassing detailed genetic, biochemical and advanced microscopy studies, has already provided a wealth of information on the identity of the key proteins and macromolecules in the divisome generally (Typas et al., 2012; Egan and Vollmer, 2013; Haeusser and Margolin, 2016; Du and Lutkenhaus, 2017, 2019; Egan et al., 2020). Moreover, the field of bacterial cell biology has undergone a renaissance recently, in particular enabled by the technique of fluorescent D-amino acid (FDAA)-based PG labelling, enabling visualisation of the coordination of events between cell division and PG biosynthesis (Kuru et al., 2012, 2019; Hsu et al., 2017, 2019). Studies have been carried out in a number of model organisms, including rod-shaped *Escherichia coli* and *Bacillus subtilis*, as well as those with particular morphological and biomedical interest (Eswara and Ramamurthi, 2017; Hsu et al., 2019). In addition, metabolic labelling has been achieved using azide-bound D-Ala-D-Ala incorporation to allow Direct Stochastic Optical Reconstruction Microscopy (dSTORM) analysis of PG synthesis with very high resolution (Trouve et al., 2021). Besides dSTORM, other microscopy advances such as the use of 3D-Structured Illumination Microscopy (3D-SIM; Tsui et al., 2014; Perez et al., 2021b) and Total Internal Reflection Fluorescence Microscopy (TIRFm; Zhao et al., 2018; Perez et al., 2019; Squyres et al., 2021) have contributed studies of division and motion in relatively small bacterial cells. Many of the key cell division proteins are highly conserved in these organisms, underlying their essentiality and universality, although their functions can vary between species.

Here, we focus on the Gram-positive, human commensal bacterium *Streptococcus pneumoniae* (pneumococcus), the classical model for bacterial transformation (Griffith, 1928) and capsule formation (Geno et al., 2015). This opportunistic human respiratory pathogen resides within the nasopharynx of a healthy individual, often without symptoms, from early after birth (Austrian, 1986). Infections occur when, in an immunocompromised or virally infected individual, pneumococcus migrates to the sterile lining of the alveoli, where it causes inflammation and activation of sputum-producing neutrophils (Weiser et al., 2018) leading to pneumonia symptoms. Once invaginated by the alveoli epithelium, the bacteria can also enter the blood stream and cross the blood-brain barrier to cause bacterial meningitis (Koedel et al., 2002), making pneumococcal infection a serious clinical issue. Pneumococcal disease is compounded by the presence of over 90 distinct strains with different capsule serotypes (Jefferies et al., 2004; Hausdorff et al., 2005), allowing pneumococcus to circumvent the actions of the currently available vaccines, which are based on a limited number of capsule serotypes (Shapiro et al., 1991; Lynch and Zhanel, 2010; Moffitt et al., 2011). This combined with the increasing prevalence of drug resistant pneumococcus [31% of worldwide cases were resistant to one or more antibiotics in 2018 (Centers for Disease Control and Prevention, 2018)] has driven the recent surge of research into pneumococcal cell division as a potential target for future chemotherapeutic strategies governing the development of next-generation antibiotics.

**FIGURE 1:**
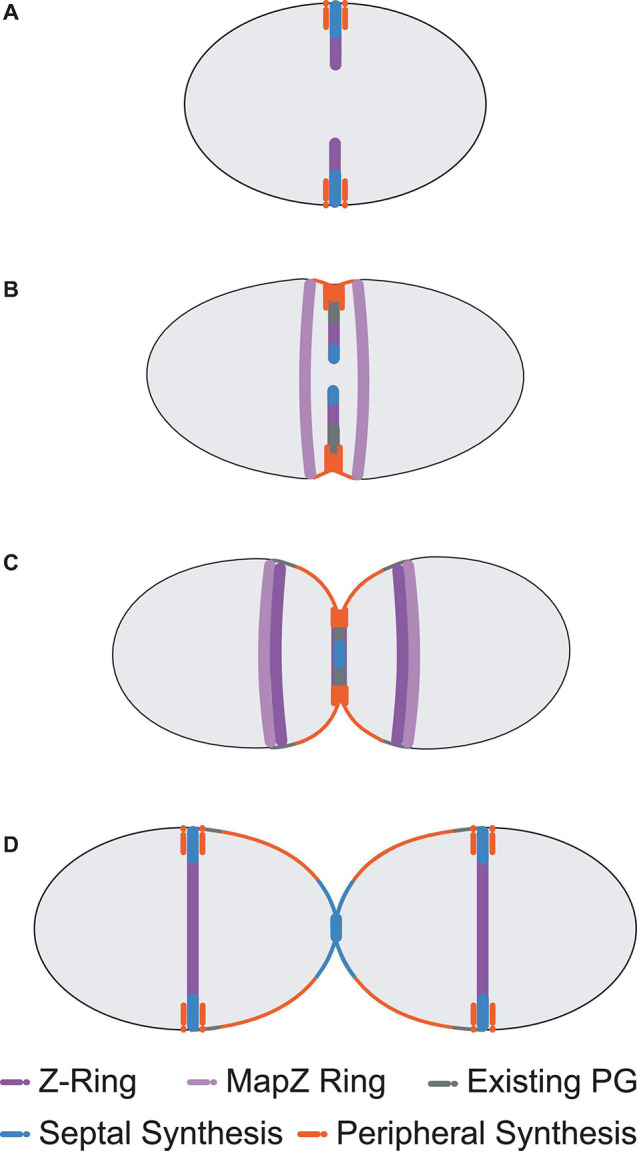
Pneumococcal cell division morphology. Following the initial formation of the Z-ring and divisome machinery, septal and peripheral PG synthetic complexes begin to make new PG as invagination begins **(A)**. Two concentric rings of newly synthesised PG surrounding the existing sacculus form as a result, whilst a portion of MapZ, FtsZ, EzrA, and FtsA (MapZ Ring) begin to migrate to the new equators. For simplicity, newly synthesised peripheral PG is drawn as a single orange colour, but likely consists of some mixture of newly synthesised peripheral PG attached to remodelled septal PG (see Trouve et al., 2021) **(B)**. Septal PG synthesis continues to close the central septum whilst peripheral PG synthesis continues to elongate the cell from midcell **(C)**. This process continues until the septum is closed. The divisome machinery then migrates to the midcells of the newly formed daughter cells and the cycle repeats **(D)**. Note that pneumococcus exists natively as encapsulated cells that are often in chains of divided cells (Barendt et al., 2009).

**FIGURE 2:**
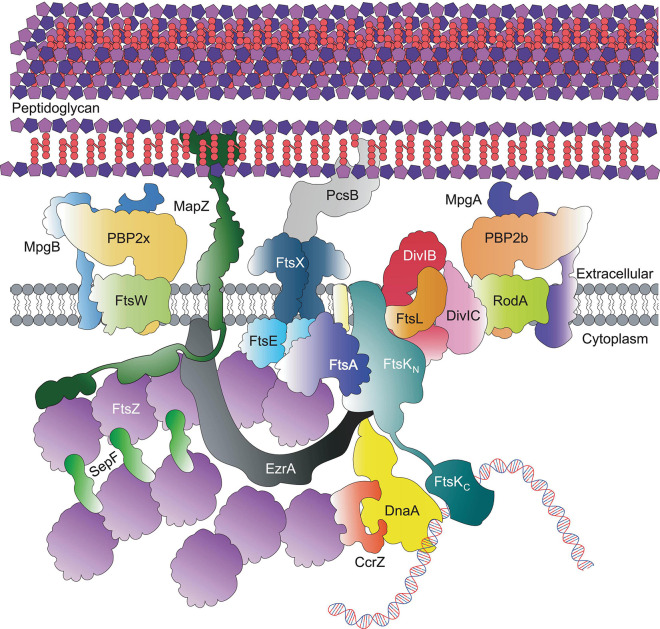
Core pneumococcal divisome components at equators of predivisional cells. For simplicity, components that localise to the equatorial ring, including Class A PBPs, the Rod complex and regulatory proteins are not shown here; but depicted in Figure 3.

**FIGURE 3:**
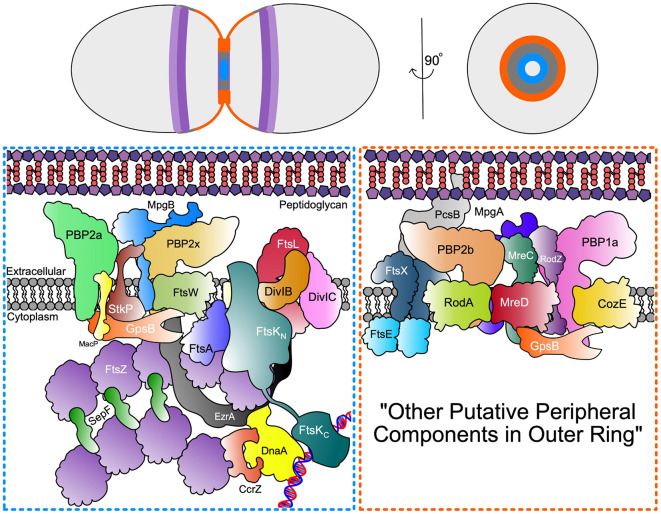
Septal and Peripheral PG synthesis components. (Top) Organisation of FtsZ Ring (dark purple) and MapZ ring (light purple) as well as septal (blue) and periphery (orange) PG synthases represented across the longitudinal and transverse plan of the pneumococcal cell. (Bottom) Schematic representation of the spatial and functional separation of divisome machinery between septal (blue box) and peripheral (orange box) machinery located in the inner and outer PG synthesis rings at opposite edges of the midcell annular disk. The organisation of the outer peripheral ring is unknown and may contain additional components, thus denoted here as “other putative peripheral components in outer ring”.

Although less well characterised than rod-shaped bacteria, many details of cell division in ovoid-shaped bacteria such as pneumococcus are known (Zapun et al., 2008b; Massidda et al., 2013; Pinho et al., 2013; Vollmer et al., 2019). The characteristic prolate ellipsoid shape is produced by coordinated PG assembly at midcell, producing the new cell-hemispheres in between old hemispheres. Initially, PG synthesis proteins are recruited to the FtsZ rings at the equator of the newly formed daughter cells at the beginning of division (Figures 1A, 2; Fleurie et al., 2014b; Tsui et al., 2014; Perez et al., 2019) and PG synthesis occurs via two separate modes, termed septal and peripheral (Hakenbeck et al., 2012; Berg et al., 2013; Land et al., 2013; Tsui et al., 2014; Straume et al., 2017). Septal synthesis produces the cell wall separating the new daughter cells, while peripheral synthesis is responsible for cell elongation (Berg et al., 2013; Tsui et al., 2014; Philippe et al., 2015; Straume et al., 2017). In pneumococcus the septal and peripheral machineries both remain at midcell throughout division, but form spatially distinct concentric rings as division proceeds, with the septal machine moving with FtsZ to the inner edge of the constricting septal annulus, whilst the peripheral machine remains in the outer ring (Figures 1B,C, 3, top; Land et al., 2013; Tsui et al., 2014, 2016; Rued et al., 2017; Sharifzadeh et al., 2017, 2020; Perez et al., 2021b). Concentric rings of newly synthesised PG consistent with this model were recently visualised by both 3D-SIM (Perez et al., 2021b) and by dSTORM (Trouve et al., 2021). As division begins, a portion of FtsZ, EzrA, and FtsA begin to migrate to the equatorial sites of the developing daughter cells, guided by MapZ (also called LocZ) (Figure 1; Fleurie et al., 2014a; Holečková et al., 2014; Perez et al., 2019). This process is discussed further below. Finally, as septum formation finishes and cell separation occurs, PG synthesis proteins migrate to the FtsZ rings at the equators of the newly formed daughter cells (Figure 1D).

This process contrasts with division in other cocci bacteria such as *Staphylococcus aureus*, which forms a divisional transverse septum spanning the axis of the cell, before “popping” open into two hemispherical daughter cells that are then able to rebuild the rest of their cocci shape (Tzagoloff and Novick, 1977; Pinho et al., 2013; Monteiro et al., 2015; Saraiva et al., 2020). This has been described as formation of a “pie crust” immediately prior to division, which has been extensively characterised using atomic force and other microscopy techniques (Turner et al., 2010; Monteiro et al., 2015; Viljoen et al., 2020). It should also be noted that *S. aureus* undergoes a much shorter elongation stage than pneumococcus, making them not truly spherical (Monteiro et al., 2015; Pereira et al., 2016; Reichmann et al., 2019). This makes pneumococcus an interesting subject of study, as even in the absence of an MreB homolog that is commonly associated with rod-shaped elongation (Land and Winkler, 2011; Philippe et al., 2014), it still exhibits a prolonged elongation phase during division (Wheeler et al., 2011), something not traditionally seen in other cocci bacteria. Although many pneumococcal division proteins are conserved in bacteria with different morphologies (such as rod-shaped *B. subtilis* or coccoid-shaped *S. aureus*) (Pinho et al., 2013), they seem to play different roles and have different spatiotemporal interactions. Moreover, the accepted notion of “sequential assembly” of the divisome components as seen in *E. coli* (Buddelmeijer and Beckwith, 2002) has not yet been demonstrated; but is still implied in *S. pneumoniae.* This article aims to update and consolidate the current understanding of the pneumococcal cell division, as outlined in Figure 1 beyond previous excellent reviews (Massidda et al., 2013; Pinho et al., 2013; Philippe et al., 2014; Vollmer et al., 2019).

## Early-Stage Assembly

In *S. pneumoniae*, the initial FtsZ-ring assembly at the equators of newly divided daughter cells organises all of the components required for cell division, septal and peripheral PG synthesis and chromosome segregation (Figure 2; Fleurie et al., 2014b; Tsui et al., 2014; Perez et al., 2019, 2021b). In this regard, the pneumococcal FtsZ-ring assembly resembles the predivisional PG complexes at the septa of rod-shaped bacteria (Buddelmeijer and Beckwith, 2002; den Blaauwen et al., 2008). Assembly of equatorial FtsZ rings begins even before division is complete, resulting in a distinctive pattern of three FtsZ rings (one at the old septum and two at the future equators) in late divisional cells (Figure 1C; Land et al., 2013; Jacq et al., 2015). In pneumococcus, the initial equatorial FtsZ-ring assembly surrounds the undivided bacterial chromosome (Tsui et al., 2014). Canonical nucleoid occlusion and Min systems found in rod-shaped bacteria are absent in pneumococcus, and replaced by the CcrZ and MapZ systems described below (Fleurie et al., 2014a; Holečková et al., 2014; Gallay et al., 2021). The initial stages of divisome assembly are broadly conserved in nearly all bacterial species. FtsZ, the homolog of eukaryotic tubulin, localises to the inner side of the cytoplasmic membrane via FtsA (Pichoff and Lutkenhaus, 2005; Mura et al., 2017). FtsZ monomers form short protofilaments at the midcell and move circumferentially around the short axis of the cell via a rapid GTP-dependent polymerisation and depolymerisation treadmilling mechanism, similar to that of eukaryotic tubulin (Dai and Lutkenhaus, 1991; Adams and Errington, 2009; Erickson et al., 2010; Meier and Goley., 2014; Haeusser and Margolin, 2016; Bisson-Filho et al., 2017; Yang et al., 2017; Perez et al., 2019). FtsZ treadmilling velocity appears to be controlled by the manner of membrane anchoring, and FtsZ GTPase activity (Bisson-Filho et al., 2017; Yang et al., 2017; Perez et al., 2019; García-Soriano et al., 2020) is important for correct divisome formation (Yang et al., 2017; Du and Lutkenhaus, 2019).

FtsA, also referred to as bacterial actin, is able to form its own filaments by binding ATP *in vitro* (Szwedziak et al., 2012). Co-localisation of FtsZ and FtsA, indicative of interaction, has been observed at all stages of cell division (Perez et al., 2019), unlike in other model bacteria, such as *E. coli* and *B. subtilis*, where inactivation of FtsA causes filamentation rather than the cell lysis seen in pneumococcus (see Mura et al., 2017). A predicted amphipathic helix at the C-terminus of FtsA is thought to lay against the membrane to facilitate its role as an FtsZ anchor (Pichoff and Lutkenhaus, 2005). ATP binding causes a conformational change at the C-terminus, which exposes the amphipathic helix and promotes membrane association and polymerisation (Krupka et al., 2014). Although FtsZ and FtsA bear homology to eukaryotic cytoskeletal components, there has as yet been no evidence to suggest the presence of motor proteins similar to myosin or kinesin. Besides simply anchoring FtsZ to cell membranes, pneumococcal FtsA seems to play regulatory roles in coordinating septal and peripheral growth at midcells (Mura et al., 2017).

Besides FtsA, EzrA, SepF, and ZapA may also play roles in anchoring pneumococcal FtsZ filaments and bundles to cell membranes. Like FtsA, EzrA is essential in *S. pneumoniae* (Thanassi et al., 2002; van Opijnen et al., 2009; Perez et al., 2021a). EzrA is a bitopic protein, whose cytoplasmic domain forms a spectrin-like coiled-coil structure (Cleverley et al., 2014). Dimers of EzrA spectrin-like molecules have been proposed to form arch structures in the cytoplasm that bind to FtsZ and modulate lateral interactions of the bundles (Cleverley et al., 2014). Along with FtsA, pneumococcal EzrA is found in treadmilling nascent FtsZ filaments and bundles that move out with MapZ toward the equators of daughter cells (Perez et al., 2019). This association argues against EzrA acting as a negative regulator that promotes FtsZ depolymerisation as reported in *B. subtilis* (Levin et al., 1999). Instead, pneumococcal EzrA is required for pneumococcal FtsZ ring formation (Perez et al., 2021a). In support of this hypothesis, *B. subtilis* EzrA, ZapA, and SepF have been shown to condense treadmilling FtsZ filaments at midcell into an FtsZ ring that can help promote cell division and septal PG synthesis (Squyres et al., 2021). In pneumococcus, SepF rings are proposed to sit perpendicular to the FtsZ filaments and group them into bundles (Singh et al., 2008; Duman et al., 2013). This plays a role in efficient cell division, as the absence of SepF results in cells displaying Z-ring constriction defects (Mura et al., 2017). Although it remains to be determined, it is likely that the pneumococcal SepF and ZapA homologs also act to bundle FtsZ filaments to form fibres, in much the same way that ZipA does in *E. coli* (Hamoen et al., 2006; Duman et al., 2013; Krupka et al., 2018). Pneumococcal ZapA is not essential (Thanassi et al., 2002) and has not yet been fully characterised, however, *B. subtilis* ZapA was shown to promote FtsZ bundle formation (Gueiros-Filho and Losick, 2002). Though it remains to be determined, it is likely that pneumococcal EzrA, SepF, and ZapA modulate the formation of FtsZ filaments during condensation of the mature FtsZ ring.

As mentioned above, pneumococcal MapZ has been shown to guide treadmilling FtsZ filaments and bundles throughout the division cycle from the septum to the equators of daughter cells (Fleurie et al., 2014a; Holečková et al., 2014; Perez et al., 2019). However, pneumococcal MapZ is not an essential protein, and in its absence, treadmilling FtsZ filaments and bundles move by a streaming failsafe mechanism imprecisely to daughter cells (Perez et al., 2019). This results in frequently misaligned FtsZ rings (Fleurie et al., 2014a; Holečková et al., 2014; Perez et al., 2019). In contrast, MapZ acts more like a beacon in *Streptococcus mutans* cells, where MapZ first moves to equators without nascent FtsZ filaments and bundles followed by streaming of FtsZ from the septum (Li et al., 2018). MapZ is a bitopic membrane protein that binds to the extracellular PG layer via its C-terminus, whilst its N-terminus associates with the cytoplasmic FtsZ (Fleurie et al., 2014a; Hosek et al., 2020). Interestingly, it appears that the C-terminal region of FtsZ is not required for its association with MapZ, but is required for associations with FtsA; the classical membrane anchor for the Z-ring (Hosek et al., 2020). At the start of division, the MapZ ring at the midcell of the predivisional cell splits into two rings on both sides of the developing septum, binds FtsZ, FtsA, and EzrA in increasing amounts, and moves toward the future equators of the daughter cells (Figure 1; Fleurie et al., 2014a; Holečková et al., 2014; Perez et al., 2019). It is postulated, but not experimentally established, that the progressive movement of the MapZ/FtsZ/FtsA/EzrA plane is driven by peripheral PG elongation synthesis. A further important consideration is the manner in which MapZ recognises the midcell and other proteins, a phenomenon which remains unknown at this point. One interesting hypothesis is that distinct regions of the lipid membrane, comprised of different lipids according to cell geometry, could serve as markers for localisation of membrane proteins such as pneumococcal MapZ (Calvez et al., 2019), however, this remains to be shown experimentally.

## Late-Stage Assembly

In *E. coli*, following the establishment of the FtsZ-ring, there is an ordered assembly of divisome component proteins starting with FtsEX (Pichoff et al., 2019). However, the order of assembly of the divisome at the equators of predivisional pneumococcal cells has not yet been established. With the exception of FtsN, homologs of the *E. coli* divisome proteins are present in *S. pneumoniae*, and it seems likely that the pneumococcal divisome assembles in the same order, which will be assumed here. Notably, at a point early after the start of division, septal and peripheral PG synthesis proteins separate into concentric rings (Perez et al., 2021b). During this separation, some proteins remain in the closing inner-ring septal PG synthesis machine, including FtsZ and PBP2x, while other proteins partition and remain in the outer-ring peripheral PG synthesis machine, including PBP2b, FtsX, and PBP2x (Perez et al., 2021b).

FtsE and FtsX are likely to be the next proteins to assemble into the nascent pneumococcal divisome. The membrane-spanning FtsX assembles into a dimer with overall predicted structural homology to the ABC transporter MacB (Leeuw et al., 1999), though it has no known role in transporting substrates; whilst the ATPase FtsE (Alcorlo et al., 2020) associates with the cytoplasmic side of FtsX. In pneumococcus, FtsEX forms a complex with the extracellular PG hydrolase, PcsB (Sham et al., 2011, 2013; Bartual et al., 2014). Like FtsX and FtsE, PcsB itself is essential in pneumococcus, as cells depleted of PcsB show misplaced newly formed cell walls, as well as partially divided cells that are still joined by their sacculus (Barendt et al., 2009; Bartual et al., 2014). Although likely to form a complex with FtsEX, based upon similarity to *E. coli* (Cook et al., 2020) and *B. subtilis* (Meisner et al., 2013) homologs, it is not yet known at which stage in cell division PcsB associates with FtsEX. Furthermore, FtsX is found in the outer peripheral PG synthesis ring as division progresses (Perez et al., 2021b), indicating that FtsEX-PcsB mediates peripheral PG elongation synthesis, analogous to FtsEX-CwlO in *B. subtilis* (Meisner et al., 2013). Thus, it is possible that FtsEX plays an early role in divisome assembly and a later role in peripheral PG remodelling. After PcsB associates with FtsEX, ATP binding and hydrolysis by FtsE is thought to provide the driving force for the mechanotransmission that activates PcsB (Sham et al., 2013; Pichoff et al., 2019; Rued et al., 2019), but how this is regulated is as yet unclear. The X-ray crystal structure of pneumococcal PcsB shows a coiled-coil domain preceding the hydrolytic CHAP domain which houses the active site Cys (Bartual et al., 2014). The PcsB coiled-coil domain interacts with the large extracellular loop domain of FtsX (Rued et al., 2019). Unlike the EnvC hydrolase of *E. coli*, PcsB is catalytically active, provided that the coiled-coil domain is removed, suggesting a control mechanism for its activity (Bartual et al., 2014; Rued et al., 2019).

Following the recruitment of the FtsEX-PcsB subcomplex, further divisome components are likely recruited in a highly ordered manner. FtsK may be another early addition, serving as a motor to separate the chromosomal DNA across the septal site (Aussel et al., 2002). Several structures of the hexameric motor domain of FtsK have been solved for the *Pseudomonas aeruginosa* protein; which describe an ATP-dependent “inchworm” mechanism of translocation in which each subunit is in one of six conformational states (Massey et al., 2006; Jean et al., 2020). The rest of this protein remains challenging to study crystallographically due to the predicted long disordered region between the polytopic membrane anchor and the motor domain. This spacer region could play a role in positioning the DNA strands exiting the motor such that each daughter cell receives a complete chromosome before septation completes. Several assays have been conducted on purified FtsK from *E. coli*, which demonstrate the speed with which this motor is able to reposition DNA, whilst also observing that the DNA sequence is sufficient only to influence FtsK directionality (Pease et al., 2005). Since pneumococcus does not possess the Min or nucleoid occlusion systems, the role of FtsK may be different from that of *E. coli* FtsK, and more analogous to SpoIIIE in *B. subtilis* sporulation (Khanna et al., 2020).

Following FtsK binding, the conserved DivIBC-FtsL subcomplex is likely recruited to the divisome. Homologous to the FtsQLB complex in *E. coli* (where DivIB is FtsQ and DivIC is FtsB), the full complex has been suggested to form in pneumococcus only during septation, despite the components being present throughout the cell cycle (Noirclerc-Savoye et al., 2005). DivIC binds FtsL via its extracellular coiled-coil domains (Masson et al., 2009) and may also have a role in stabilising FtsL (Sievers and Errington, 2000; Wadenpohl and Bramkamp, 2010), though its exact function in many organisms remains unclear. In *B. subtilis*, DivIC reportedly protects FtsL against RasP cleavage by shifting the oligomeric state of FtsL to a dimeric form (Wadenpohl and Bramkamp, 2010). Given that the homologous protease RseP from *E. coli* uses zinc as a co-factor (Hizukuri et al., 2017), it is possible that zinc availability may be an important and unexplored regulator of this stage of pneumococcal cell division. FtsL is essential in *E. coli* and is thought to play a role in zinc sensitivity and by extension, membrane permeability (Guzman et al., 1992; Blencowe et al., 2011). Although the role of pneumococcal FtsL is unknown, the conservation of this complex across all bacteria makes it likely that FtsL’s function is also conserved. The membrane spanning protein DivIB has also been shown to interact with DivIC-FtsL via its central β-domain, and is not essential for growth unlike FtsQ in *E. coli*; with deletion of the gene leading to long chains of pneumococcal cells, as well as cells with impaired septa in rich media (Gouëllec et al., 2008). FtsL is rapidly degraded in the absence of DivIB, suggesting that the latter serves to stabilise the former, either physically or by other means (Gouëllec et al., 2008). Interestingly, the full DivIBC-FtsL subcomplex only co-localises in pneumococcus during septation (Noirclerc-Savoye et al., 2005). DivIB and FtsL localise to the midcell only during septation, whereas DivIC follows the localisation pattern of the FtsW-PBP2x complex, residing always at the septal site until late in division (Noirclerc-Savoye et al., 2005). This could indicate that the function of the DivIBC-FtsL complex at the site of division is controlled, at least in part, by DivIC; and that DivC is involved in the recruitment of the PG synthesis subcomplex FtsW-PBP2x. It has also been shown in other bacteria that the DivIBC-FtsL subcomplex homologs are involved in the regulation of PG synthases (Boes et al., 2019; Marmont and Bernhardt, 2020).

At some point in the assembly of the divisome, the newly discovered protein CcrZ interacts with FtsZ and couples cell division to DNA replication in pneumococcus and other *Firmicutes* (Gallay et al., 2021). At the midcell, the origin of replication is bound by DnaA, which CcrZ stimulates to start replication, after which the newly replicated origins segregate to daughter cells. CcrZ remains at the septum with the replication machinery throughout division and moves to the equatorial FtsZ rings of daughter cells only after replication is complete. In this way, CcrZ stimulates new rounds of replication when replicated chromosomes are correctly positioned, thereby preventing guillotining of chromosomes (Gallay et al., 2021). This mechanism ensures that DNA replication occurs just a single time in the cell cycle.

## Final-Stage Assembly

In the final phase of divisome assembly, it is crucial for the cell to start producing more PG alongside the membrane extension so that the overall shape and rigidity of the daughter cells can be maintained. In pneumococcus, there are two major PG synthase complexes that facilitate this process: FtsW-PBP2x and RodA-PBP2b, which mainly carry out septal and peripheral PG synthesis, respectively (Berg et al., 2014; Tsui et al., 2014; Perez et al., 2019, 2021b). PBP2x, however, was recently shown to remain partially localised to the periphery of the septum during constriction, suggesting that it might participate in enlargement of the septal annular ring or in peripheral PG synthesis (Perez et al., 2021b). Each of these PG synthase complexes consists of a shape, elongation, division, sporulation (SEDS) family glycosyltransferase (FtsW; RodA) with a cognate Class B PBP (PBP2x; PBP2b) (Gérard et al., 2002; Fraipont et al., 2011; Zapun et al., 2012; Sjodt et al., 2018, 2020). The FtsW-PBP2x complex migrates circumferentially around the septal FtsZ ring leading to invagination between daughter cells (Perez et al., 2019). FtsW-PBP2x complex movement is driven by septal PG synthesis itself and is not directly dependent on treadmilling of FtsZ filaments and bundles (Perez et al., 2019).

As in other bacteria (den Blaauwen et al., 2008; Rohs et al., 2018), pneumococcal RodA-PBP2b is part of an elongasome “Rod” complex, which in pneumococcus, contains several regulatory and organising proteins, including MreC, MreD, and RodZ, but not MreB (Land and Winkler, 2011; Land et al., 2013; Philippe et al., 2014; Tsui et al., 2016; Stamsås et al., 2017; Straume et al., 2017; Zheng et al., 2017; Winther et al., 2021). This complex in pneumococcus carries out a form of sidewall synthesis that pushes new peripheral PG out from an outer ring at the midcell, and participates in cell elongation (Figure 3; Tsui et al., 2014; Perez et al., 2021b; Trouve et al., 2021). Several investigations show that septal and peripheral PG synthesis occur concurrently throughout most of the pneumococcal cell cycle, instead of as separate elongation and septal-closure phases (Wheeler et al., 2011; Tsui et al., 2014; Perez et al., 2021b; Trouve et al., 2021). Although septal and peripheral PG synthesis occur separately and are catalysed by distinct synthases (Tsui et al., 2014; Perez et al., 2021b; Trouve et al., 2021), protein interaction profiles and phenotypes of mutants lacking certain proteins, such as those for GpsB (Rued et al., 2017; Cleverley et al., 2019), suggest mechanisms for coordination of septal and peripheral PG synthesis to give final cell shapes and sizes. Another important regulatory protein, DivIVA, seems to mediate division, cell morphology (possibly through PG peripheral synthesis), and chromosome segregation in *S. pneumoniae* (Vollmer et al., 2019); however, since its exact function remains unknown (Fadda et al., 2007; Fleurie et al., 2014b; Straume et al., 2017), it is not included in Figure 3.

Besides the Class B enzymes, PBP2x and PBP2b, mediators of septal and peripheral PG synthesis, respectively, the Class A PBPs, PBP1a, PBP2a, and PBP1b, also play roles in PG synthesis and PG repair in pneumococcus [recently reviewed in Straume et al. (2021)]. Unlike the Class B PBPs, Class A PBPs are bifunctional enzymes and contain a glycosyltransferase (GT) and transpeptidase (TP) domain in the same polypeptide chain separated into distinct domains (Straume et al., 2021). Like most bacteria, pneumococcus contains a pair of Class A PBPs, PBP1a, and PBP2a, whose simultaneous deletion results in a synthetic lethal phenotype (Straume et al., 2021). Mutants lacking PBP2a or PBP1b generally show minimal cell morphology defects in culture, whereas mutants of the progenitor D39 serotype 2 strain lacking PBP1a form narrower, slightly longer cells under some culture conditions (Land and Winkler, 2011). Genetic suppression patterns strongly implicate PBP1a in peripheral PG synthesis (Land and Winkler, 2011; Tsui et al., 2016). In addition, PBP1a localises in rings at the midcell in the same patterns as PBP2b and MreC, which mediate peripheral PG synthesis (Figure 3; Tsui et al., 2014). In contrast, Class A PBPs that mediate sidewall elongation of rod-shaped bacteria localise diffusely over the bodies of cells (Rohs et al., 2018). High-level penicillin resistance in pneumococcus is due to mutagenic alterations in the *pbp2x*, *pbp2b*, and *pbp1a* genes (Barcus et al., 1995; Chesnel et al., 2005; Zapun et al., 2008a; Hakenbeck et al., 2012).

Consistent with a role in peripheral PG synthesis, PBP1a interacts with the polytopic membrane protein CozE, a member of the MreCD-Rod complex, which directs PBP1a to the midcell elongasome (Fenton et al., 2016). A *S. aureus* CozEb paralog of pneumococcal CozE has also recently been reported and suggested to play a role in cell-shape homeostasis (Stamsås et al., 2018). Much less is known about the function of PBP2a and PBP1b in pneumococcus. PBP2a activity was reported to be activated by phosphorylated MacP protein (Fenton et al., 2018). MacP is phosphorylated by the Serine/Threonine protein kinase StkP, which interacts with and phosphorylates a number of proteins involved in pneumococcal cell division and PG synthesis (Fleurie et al., 2012, 2014b; Grangeasse, 2016; Manuse et al., 2016). Besides phosphorylated MacP, PBP2a directly interacts with the regulatory protein GpsB (Cleverley et al., 2019) which also positively regulates the levels of StkP-mediated protein phosphorylation (Fleurie et al., 2014b; Rued et al., 2017). It has been postulated that phosphorylation of MacP by StkP activates PBP2a in septal PG synthesis (Fenton et al., 2018), however, more generally, the roles of protein phosphorylation in pneumococcus remain unclear; though likely important. Problematically, phosphoablative and phosphomimetic mutants of phosphorylated cell division and PG synthesis proteins do not uniformly show phenotypes in exponentially growing cultures (Fleurie et al., 2012; Grangeasse, 2016; Manuse et al., 2016; Zheng et al., 2017); suggesting other factors such as genetic background, culture conditions, growth phase, and/or cell stress may modulate the phenotypic effects of protein phosphorylation.

The recent discovery of primary PG synthases consisting of Class B PBPs and cognate SEDS proteins (Taguchi et al., 2019; Sjodt et al., 2020), has raised the question of whether Class A PBPs play direct roles in PG synthesis (Straume et al., 2020, 2021; Pazos and Vollmer, 2021). In support of this, there is strong evidence that Class A PBPs interact with cell division and PG synthesis proteins and account for a considerable amount of PG synthesis in exponentially growing, non-stressed cells (Pazos and Vollmer, 2021; Straume et al., 2021). However, recent results in *E. coli* and *S. pneumoniae* implicate Class A PBPs in PG repair in cells subjected to cell wall stresses (Straume et al., 2020; Vigouroux et al., 2020). In pneumococcus, inhibition of the septal synthetic complex FtsW-PBP2x results in the cells becoming resistant to cleavage by exogenously added PG hydrolase CbpD (Straume et al., 2020). Under the conditions used here, only PBP2x was being inhibited by oxacillin. When combined with other localisation studies showing CbpD binding to the septal region (Eldholm et al., 2010), a remodelling role for the Class A PBPs can be suggested; one which alters the crosslinking of septal PG strands to protect the sacculus from degradation by CbpD (Straume et al., 2020).

As noted in two recent reviews, the roles of Class A PBPs in normal PG synthesis and in PG repair of damaged cell wall during stress conditions are not mutually exclusive (Pazos and Vollmer, 2021; Straume et al., 2021). In this regard, an interesting hypothesis (Straume et al., 2021) was recently put forward that during septal and peripheral PG synthesis, pneumococcal Class A PBPs act in concert to the Class B PBP-SEDS synthases to lay down a separate internal layer of PG, characterised by a dense PG mesh of randomly orientated strands (Pasquina-Lemonche et al., 2020). In the septal division plane, this disordered internal layer is built on top of the ordered concentric rings of PG strands synthesised by Class B-SEDS synthases, such as PBP2x-FtsW, in pneumococcus and other *Firmicutes* (Pasquina-Lemonche et al., 2020; Straume et al., 2021). Whether this internal layer of PG is synthesised primarily by Class A PBPs alone as postulated, or results from the extensive remodelling of ordered PG synthesised by a combination of Class B PBP-SEDS and Class A PBPs remains to be determined.

Remodelling of PG by hydrolases is required to release nascent glycan strands from their lipid anchors and to cleave amide bonds in muropeptides to allow integration of newly synthesised peptidoglycan strands (Massidda et al., 2013; Vollmer et al., 2019). A recent model suggests that remodelling of septal PG and integration with newly synthesised peripheral PG occurs throughout the cell division cycle (Trouve et al., 2021). According to this model, the final peripheral PG then consists of a patchwork of PG synthesised by both machines: septal PG at the outer edge of the septal annulus is cleaved by PG hydrolases in a concerted manner, and woven into the newly synthesised peripheral PG. Consistent with this model, PBP2x and PBP2b arrive concurrently at new division sites (Tsui et al., 2014), inhibition of PBP2x alters cell shape in pre-divisional cells (Philippe et al., 2015), and concentric PG synthesis rings are observed at very early stages of the cell cycle (Perez et al., 2021b; Trouve et al., 2021).

However, the pulse-chase PG labelling data used to support this model can alternatively be interpreted to indicate that peripheral PG synthesis starts before septal PG synthesis in newly divided cells. This alternative interpretation was also proposed in a previous study showing that in terms of geometry, cell elongation precedes separation in ovococci bacteria (Wheeler et al., 2011). Further studies are needed to determine the timing and extent of remodelling at the junction of septal and peripheral PG. A leading candidate for a remodelling endopeptidase in this process is the essential FtsEX-PcsB discussed above, which localises to the outer peripheral synthesis ring of the septal annulus (Perez et al., 2021b). Other PG endopeptidases that participate in pneumococcal PG remodelling are currently unknown, but antibiotic and cell wall stress conditions induce the transcription of the WalRK regulon, which includes PcsB as well as putative PG binding proteins of unknown functions (Ng and Winkler, 2004; Ng et al., 2005).

Two other PG hydrolases have been identified that release glycan chains from lipid precursors in the separate septal and peripheral PG synthesis nanomachines. Lack of MpgB [previously PMP23 (Jacq et al., 2018)] leads to aberrant localisation of divisome proteins as well as septal defects, whilst MpgA (previously MltG*^*Spn*^*) localises to and is linked genetically to the peripheral PG synthesis machinery (Tsui et al., 2016). A new paper demonstrates that both MpgB and MpgA are muramidases with different points of glycan chain cleavage (Taguchi et al., 2021). MpgB cleaves nascent peptidoglycan at the MurNAc-GlcNAc closest to the lipid anchor. In contrast, MpgA shows homology to *E. coli* MltG, including a LysM domain that specifies the cleavage site such that the MpgA cleaves after every seventh dipeptide from the lipid anchor at the periphery (Taguchi et al., 2021). Whilst not essential, the mutant MpgA lacking the LysM domain is not fully functional in pneumococcal cells (Taguchi et al., 2021), therefore the separation between septal and peripheral PG synthesis machinery is also seen for remodelling PG hydrolases, resulting in a different biochemical environment at each site. Finally, unlike in *E. coli* and *B. subtilis* where about 50% of PG is turned over and recycled per generation during growth, there is minimal turnover of “old” PG by pneumococcus during planktonic growth and in host-relevant biofilms (Boersma et al., 2015). Minimal release of PG breakdown products from turnover may be a strategy that pneumococcus uses to avoid alerting the host innate immune system to its presence.

## Other Division Considerations

It should also be noted that, despite sharing some morphology events with rod-shaped bacteria, Gram-positive bacteria, including pneumococcus, do not encode an FtsN homolog found in Gram-negative organisms. In *E. coli*, this essential protein is known to interact with PBP1b, PBP3 and the FtsQLB complex (Müller et al., 2007; Liu et al., 2015), but also with FtsA, which is able to recruit FtsN to the septal site and allow it to activate PBP3 (Busiek and Margolin, 2014). This protein appears to act as a kind of checkpoint for septal constriction, since only after the binding of FtsN does septal PG synthesis appear to begin (Liu et al., 2015). This raises the interesting question of whether there is an equivalent control mechanism in Gram-positive organisms such as pneumococcus. At present, this is a question that remains unanswered. Moreover, the relationship between FtsZ treadmilling and the movement of septal PG synthases show distinct differences in *E. coli* (Yang et al., 2021), *B. subtilis* (Bisson-Filho et al., 2017), and *S. pneumoniae* (Perez et al., 2019), along with numerous similarities (McCausland et al., 2021).

Another important area that is not well understood is the effect of the extracellular capsule on pneumococcal cell division. As noted above, the pneumococcal pangenome specifies over 90 different capsular polysaccharides in the different serotype strains of *S. pneumoniae*. In addition, non-encapsulated pneumococcus strains are emerging as pathogens (Bradshaw and McDaniel, 2019). When present, the capsule is the single most important virulence factor of *S. pneumoniae*, allowing avoidance of opsonisation by phagocytotic cells of the innate immune system (Weiser et al., 2018). Multivalent capsule-based adult and conjugated vaccines provide significant protection against pneumococcal infections (Lynch and Zhanel, 2010; Moffitt et al., 2011). Depending on the serotype, the capsular polysaccharides may be linked to the membrane, covalently bound to the cell wall, or fully ejected into the extracellular space (Sørensen et al., 1990; Cartee et al., 2005); all of which could potentially provide different mechanisms for their involvement in cell division. Links have also been made between virulence and the roles of teichoic acids in *S. pneumoniae* (Vollmer et al., 2019), though any specific involvement of these crucial cell wall components and pneumococcal division has as yet not been demonstrated. However, a recent report shows that the degree and location of external modifications of PG-bound polyrhamnose of *S. mutans*, which lacks teichoic acids, controls PG hydrolase activity and placement of the axis of cell division (Zamakhaeva et al., 2021). It remains to be determined whether other ovococcal species like *S. pneumoniae* use undermodification of wall teichoic acids or exopolysaccharides to cue division (Winkler, 2021).

In this regard, presence of the serotype 2 capsule, which is covalently linked to the PG of the progenitor D39 strain, reduces the phenotypes observed for mutants defective in cell division or PG synthesis compared to an isogenic unencapsulated derivative (Barendt et al., 2009). This phenotype dampening is general and not confined to specific steps in division or PG synthesis. The mechanisms underlying phenotype dampening by the serotype 2 capsule are not understood and could involve physical constraint by the capsule that changes the timing of division or regulatory mechanisms analogous to the recently described RocS system that coordinates proper chromosome segregation and division with capsule biosynthesis (Mercy et al., 2019).

## Concluding Remarks

Pneumococcal cell division is a complex and dynamic process of distinct microbiological and biomedical importance. Cell division and PG synthesis of ovoid-shaped pneumococcus have emerged as models, with distinct mechanisms and components compared to rod-shaped and spherical model bacteria. Dynamic interactions amongst the protein components that form the broader divisome result in the formation and dissolution of many subcomplexes that facilitate the intricate morphological and biochemical changes inherently essential to the division process. This dynamic process has recently become clearer through an elegant combination of genetic and biochemical approaches, augmented by novel cell wall labelling and high-resolution microscopy studies. At its core, cell wall PG must be broken, remodelled, and resynthesised by these protein complexes in a precise order whilst also coordinating with the membrane synthesis to continue providing structure to the cell.

## Author Contributions

NB drew the figures. All authors wrote and contributed to the manuscript. MW and DR coordinated preparation of the final manuscript.

## Conflict of Interest

The authors declare that the research was conducted in the absence of any commercial or financial relationships that could be construed as a potential conflict of interest.

## Publisher’s Note

All claims expressed in this article are solely those of the authors and do not necessarily represent those of their affiliated organizations, or those of the publisher, the editors and the reviewers. Any product that may be evaluated in this article, or claim that may be made by its manufacturer, is not guaranteed or endorsed by the publisher.
